# The funding and use of high-cost medicines in Australia: the example of anti-rheumatic biological medicines

**DOI:** 10.1186/1743-8462-4-2

**Published:** 2007-03-01

**Authors:** Christine Y Lu, Kenneth M Williams, Richard O Day

**Affiliations:** 1 Faculty of Medicine, University of New South Wales, Australia, and Department of Clinical Pharmacology and Toxicology, St Vincent's Hospital Sydney, Australia

## Abstract

**Background:**

Subsidised access to high-cost medicines in Australia is restricted under national programs (the Pharmaceutical Benefits Scheme, PBS, and the Repatriation Pharmaceutical Benefits Scheme, RPBS) with a view to achieving cost-effective use. The aim of this study was to examine the use and associated government cost of biological agents for treating rheumatoid arthritis over the first two years of subsidy, and to compare these data to the predicted outcomes.

**Methods:**

National prescription and expenditure data for the biologicals, etanercept, infliximab, adalimumab, and anakinra were collected and analysed for the period August 2003 to July 2005. Dispensing data on biologicals sorted by the metropolitan, rural and remote zones and by prescriber major specialty were also examined.

**Results:**

A total of 27,970 prescriptions for biologicals was reimbursed. The government expenditure was A$53.1 million, representing only 19% of that expected. Almost all prescriptions were reimbursed by the PBS (98%, A$52 million) and the remainder by the RPBS. Approximately 62% of the prescriptions were for concessional patients (A$32.9 million). There was considerable variability in the use of biologicals across Australian states and territories, usage roughly correlating with the *per capita *adjusted number of rheumatologists. The total number of prescriptions continued to increase over the study period. Etanercept was the most highly prescribed agent (74% by number of prescriptions), although its use was beginning to plateau. Use of adalimumab increased steadily. Use of infliximab and anakinra was considerably lower. The resultant health outcomes for individual patients are unknown. Prescribers from capital cities and other metropolitan centres provided a majority of prescriptions of biologicals (89%).

**Conclusion:**

The overall uptake of biologicals for treating rheumatoid arthritis over the first two years of PBS subsidy was considerably lower than expected. Long-term safety concerns and the expanded clinical uses of these drugs emphasise the need for evaluation. It is essential that there is comprehensive, ongoing analysis of utilisation data, associated expenditure and, importantly, patient outcomes in order to enhance accountability, efficiency and equity of policies that allocate substantial resources to subsidising national access to high-cost medicines.

## Background

Expenditure on pharmaceuticals has increased rapidly in many countries in the last decade [1]. Access to medicines is increasingly problematic for healthcare systems and individuals as healthcare resources are finite and innovative medicines expensive. Critical but conflicting issues under discussion are: which medicines should be subsidised; who should have access to such drugs; what restrictions should apply to access; and what is a reasonable distribution of that cost between government and the individual patient? Arrangements for access to medicines need to be managed well so that they are acceptable to consumers, clinicians, the government and the industry. The Pharmaceutical Benefits Scheme (PBS) is Australia's drug reimbursement system that has supported national access to a wide range of medicines for the community since the 1950s. This system has attracted considerable attention worldwide as Australian drug prices are markedly lower than those in comparable countries, although prices paid for innovative medicines are similar [2]. The PBS is an uncapped scheme. The fiscal sustainability of the PBS has been under intense scrutiny by federal health and financial public policy makers in recent years because government outlays on subsidised prescription medicines have continued to increase at a rate greater than other areas of health care [3]. In part, this disproportionate expenditure reflects the cost of the new biotechnology-derived drugs. The challenge posed by increasing expenditure on pharmaceuticals and demand for innovative expensive medicines will be even more prominent in the future. Controlling government expenditure on the PBS while maintaining social equity and access to 'essential medicines' is at the centre of an ongoing public debate. Careful examination of the recent developments in targeting access to high-cost medicines in Australia is instructive in informing the debate concerning the principles and processes that might underpin appropriate and ethical access to expensive medicines under the PBS or similar reimbursement systems.

The Pharmaceutical Benefits Advisory Committee (PBAC), an independent committee consisting of clinical experts, health economists, and a consumer representative, recommends to the Federal Minister for Health which medicines should be 'listed' on the PBS. The PBAC decisions are based on an evaluation of comparative efficacy and safety as well as cost-effectiveness of medicines (including quality-adjusted life-years) [4]. Australia was the first country to introduce a mandatory requirement for economic analysis to select pharmaceuticals for a publicly funded formulary in 1993 [5]. Use of economic evaluation to inform decisions about reimbursing drugs has been adopted globally, including countries such as the United Kingdom, Canada, and New Zealand [6-10]. The PBS decisions have a direct budgetary impact and PBAC is responsible for recommending drug prices [11]. The PBS has established complex controls to balance the benefits, risks, and costs associated with high-cost medicines by targeting access to subsets of patients. PBS restrictions are based on data (primarily from randomised controlled clinical trials), economic evaluation (an assessment of 'value for money' taken from a long-term perspective), and more recently and innovatively, a collaboration between the stakeholders (the PBAC, the respective pharmaceutical companies, and representative medical specialists) [12,13]. To gain subsidised access, patients must meet specific eligibility criteria for both starting (severe disease inadequately controlled by existing cheaper treatments) and continuing therapy (substantial clinical improvement). Prescribing rights are limited to specialist physicians. These prior-approval requirements have been applied to control access to a number of high-cost medicines under the PBS. The concept of "high-cost medicines" has not yet been clearly defined internationally. In Australia, a broad definition of a high-cost medicine is one whose acquisition cost is greater than A$10,000 per patient per treatment course [14]. A representative example is seen with the biological response modifiers for the treatment of rheumatoid arthritis (RA) namely the tumour necrosis factor-alpha inhibitors (TNFIs), etanercept, infliximab, and adalimumab, and an interleukin-1 receptor antagonist, anakinra (about A$20,000 per patient per year). Subsidised access to other high-cost medicines, such as imatinib for the treatment of patients with chronic myeloid leukaemia (about A$45,000 per patient per year), is similarly restricted under the PBS. Self-funding by most patients is not a realistic option for these high-cost medicines.

The issue of subsidised access to medicines for the treatment of RA and other autoimmune diseases came into focus with the introduction of these biologicals. These drugs markedly reduce disease activity and slow erosion of joints in the majority of patients with RA [15] but they are substantially more expensive than conventional disease-modifying anti-rheumatic drugs (DMARDs). A risk-mitigation arrangement between the government and the respective sponsors established an agreed annual ceiling for government outlays [12]. An 'interchangeability rule' was later introduced (December 2004) that allowed patients with RA to trial an alternate biological without the need to re-qualify against the initial eligibility criteria [16]. Details of the controlled access scheme for these medicines under the PBS are provided in Table 1.

**Table 1:** Access scheme for biological agents for the treatment of RA under the PBS

**Main features**	**Prior-approval requirements**
**Criteria for initiating treatment**	• Severe active disease: a) elevated levels of anti-inflammatory markers (ESR > 25 mm/hour or CRP > 15 mg/L) b) swollen and tender joints – a total of > 20 joints, or > 4 major joints (elbow, wrist, knee, ankle, shoulder, hip)
	• A record of rheumatoid factor positive status (this requirement is removed as of June 2005)
	• Failure to achieve adequate response to a step-up sequence of treatment with conventional DMARDs: a) monotherapy with methotrexate (20 mg per week) b) a combination of methotrexate (> 7.5 mg per week) and 2 other DMARDs for at least 3 months c) leflunomide, leflunomide with methotrexate, or cyclosporin for at least 3 months
	• Evidence of intolerance or contraindication to DMARDs
	• Patients required to sign a 'patient acknowledgement form'
	• Treatment is approved for 16 weeks only (treatment of 22 weeks is approved for infliximab)
**A patient agreement process**	• A patient acknowledgement form to be signed by patients with their physicians to acknowledge that PBS-subsidised treatment will only continue if the predetermined response criteria are achieved at 12 weeks
**Criteria for continuing treatment**	• Clinical outcomes are evaluated according to predetermined quantifiable criteria at 12 weeks: a) Reduction in levels of anti-inflammatory markers, ESR < 25 mg/hour, or CRP < 15 mg/L, or 20% from baseline levels b) Reduction in the total number of joint count by 50%
**'Interchangeability' (introduced December 2004)**	• Patients approved to commence PBS-subsidised biological treatment are allowed to switch to an alternate biological agent at any time (an agent that the patient has responded adequately or has not trialed previously)
**Restricted prescribing rights**	• Prescription only by specialist rheumatologists initially. Prescribing rights were extended to clinical immunologists with expertise in the management of rheumatoid arthritis as of February 2004
**'Risk-mitigation' arrangement **	• Annual PBS expenditure for the TNF inhibitors group was predicted to be up to A$140 million
	• Expenditure above this figure to be covered by the sponsoring pharmaceutical companies (details not clear from public documents)

Abbreviations:

ESR = erythrocyte sedimentation rate

CRP = C-reactive protein

DMARDs = disease-modifying anti-rheumatic drugs

Accurate prediction of pharmaceutical expenditure is critical if the PBS is to be sustained and limited resources allocated appropriately. As part of major submissions to the PBAC, sponsoring pharmaceutical companies are required to estimate the uptake of the medicine and the financial implications for at least the first 2 years [13]. These estimates are based on the prevalence of the disease (chronic conditions) or the annual incidence (acute conditions) as well as the likely market share of the new medicine [17]. Submissions to the PBAC for drug subsidy containing these estimates are bound by the Australian National Health Act (1953) and the data are treated as 'commercial-in-confidence', therefore only limited information is made available to the public [11].

Approximately 1% of the Australian population has RA (~200,000 Australians) [18], and it was estimated that approximately 4,000 of these patients (i.e. approximately 2% of RA patients) would meet the proposed PBS criteria for etanercept treatment in the first year of subsidy [19]. However, this estimate is substantially below others. For example, it was estimated that about 5–6% of patients with RA would qualify for a TNFI based on the inclusion criteria for the Anti-Tumour necrosis factor Trial in Rheumatoid Arthritis with Concomitant Therapy (ATTRACT) or using criteria established by the British Society of Rheumatology [20,21]. The PBS expenditure for biologicals in the treatment of RA was forecast to be up to A$140 million per annum ("cap") at the time that subsidy of etanercept and infliximab was approved (2003). The basis for these projections was not publicly available. It is also unclear from documents available in the public domain whether there have been any alterations to the original forecast expenditure resulting from the subsequent PBS subsidy of adalimumab and anakinra in 2004. Utilisation of biologicals under the Repatriation Pharmaceutical Benefits Scheme (RPBS) was not included explicitly in the projections. The RPBS provides pharmaceutical benefits to veterans and eligible dependants that in general conform to the same requirements as for the PBS.

There is limited data on comparison of estimated and actual drug utilisation in Australia, and no study of utilisation of biologicals under the RPBS has yet been published. The aim of this study was to examine the use and government cost of biological agents for treating RA in Australia, under the PBS and RPBS, over the first two years of government subsidy (August 2003 to July 2005). Examination of these data is important to assess the implications of controlled access schemes for medicines. Findings will be helpful in guiding policy and practice on managing access to high-cost medicines under drug reimbursement systems such as the PBS.

## Methods

This study was a retrospective analysis of data on the national utilisation and expenditure for etanercept, infliximab, adalimumab, and anakinra under the PBS and RPBS for the period August 2003 to July 2005.

Medicare Australia (formerly the Health Insurance Commission), a government statutory authority that administers the PBS and other health programs nation-wide, maintains an electronic database of claims for subsidised medicines. Statistical data on reimbursement of prescriptions and expenditure were obtained from this administrative database [22]. Relevant PBS item numbers were [16]:

• Etanercept (25 mg × 8 vials/prescription): 8637N (initial treatment), 8638P (continuing treatment)

• Infliximab (100 mg, quantity supplied on the basis of the weight of the patient, at a dose of 3 mg/kg for a single infusion): 6397Q

• Adalimumab (40 mg × 2 injections): 8737W (initial treatment), 8741C (continuing treatment)

• Anakinra (100 mg × 28 injections): 8773R (initial treatment), 8774T (continuing treatment)

Methods used to interpret data from Medicare Australia have been described elsewhere [23]. In brief, the data comprised the number of 'services' (number of prescriptions) reimbursed by Medicare Australia and the government expenditure on these prescriptions. Patients paying privately, or funded by private health insurance, are not captured by the claims data. Patients must hold a valid Medicare card in order to obtain PBS-subsidised medicines and make a co-payment of A$28.60 per prescription (A$4.60 for concessional patients; 2005 rate). Medicare Australia reimburses pharmacies the difference between the co-payment made by the patient and the cost of each prescription pack of the medicine. The maximum contribution for biologicals for the treatment of RA by Medicare Australia was A$1888/prescription (four-week supply for biologicals, except infliximab which is repeated every 6–8 weeks). Data available through Medicare Australia's website are updated monthly in aggregates and can be separated by States and Territories, and by patient categories e.g. general, concessional (including senior and pensioner concessions), and veterans. Although usage in public hospitals is not captured (separately managed by state governments), treatment of RA with these agents is largely undertaken in ambulatory private practice in Australia; except infliximab which is supplied through public and private hospitals under the Highly Specialised Drugs Program [24].

In order to examine any association between access to rheumatologists and the uptake of biologicals, prescription data were adjusted for State population numbers obtained from the census data from the Australian Bureau of Statistics [25]. The number of rheumatologists in each state was obtained from the Australian Rheumatology Association (as of November 2005). Information on the proportion of full-time versus part-time rheumatologists, and whether they worked primarily in private practice or in the public system is not documented by the Association.

Dispensing data were also obtained from the Drug Utilisation Sub-Committee (DUSC) of the PBAC to examine the geographical dispensing trends. The DUSC database contains information on all subsidised prescriptions processed by Medicare Australia under the PBS and RPBS. The DUSC database also contains estimates of the number of non-subsidised (private and below co-payment) prescriptions from an ongoing survey in which a sample of community pharmacies provides records of all dispensed prescriptions – costs of these non-subsidised prescriptions are not available [26]. Aggregated prescription data on biologicals sorted by the Rural, Remote and Metropolitan Area (RRMA) classification system [27] and by prescriber major specialty were examined.

In using these databases to quantify drug usage, the assumptions are that dispensing is a good proxy for 1) prescribing (i.e. most patients prescribed a biological actually have it dispensed), and 2) consumption (i.e. adherence with these agents is likely to be high). These are reasonable assumptions for a chronic disease like RA, and because of the costs of these medicines, and complex process that prescribers and patients have to experience in order to gain access.

## Results

### Prescriptions of biologicals under the PBS and RPBS

There was a total of 27,970 prescriptions reimbursed by Medicare Australia between August 2003 and July 2005 for the biologicals used to treat RA (etanercept, 20,742; infliximab, 851; adalimumab, 6,257; anakinra, 120). The expenditure on biologicals increased steadily over the study period; a total government expenditure of A$53.1 million – 98% by the PBS (A$52 million) and the remainder (A$1.1 million) by the RPBS. The estimated patient contribution was A$267,000 over the study period (0.5% of a total cost of A$53.4 million). Under the PBS, approximately 62% of the prescriptions (17,330 prescriptions) went to concessional patients at a cost of A$34 million, and 36% to general patients at a cost of A$19.1 million.

**Figure 1:**
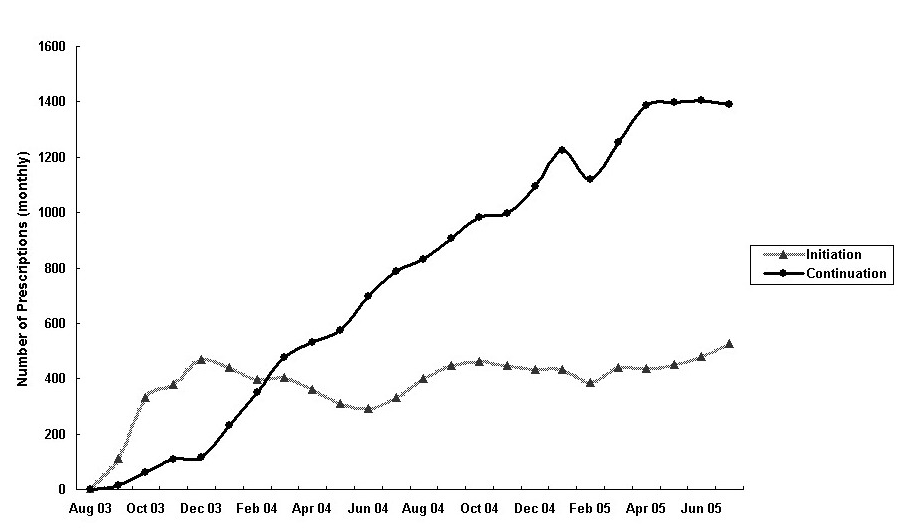
Monthly number of prescriptions for biologics under the PBS and RPBS, by initiation and continuation of therapy, August 2003–July 2005. Note: Biologics included: etanercept, adalimumab, and anakinra.

**Figure 2:**
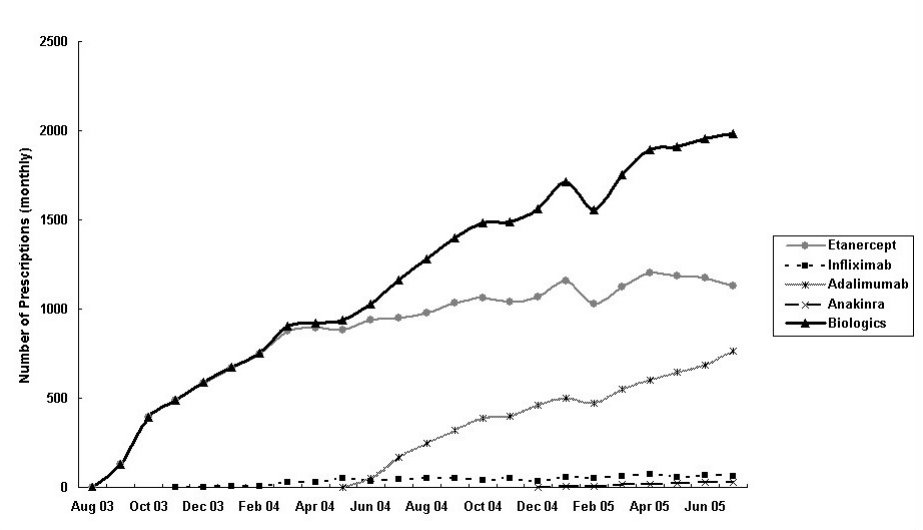
Monthly number of prescriptions for biologics under the PBS and RPBS, by drug, August 2003–July 2005.

**Figure 3:**
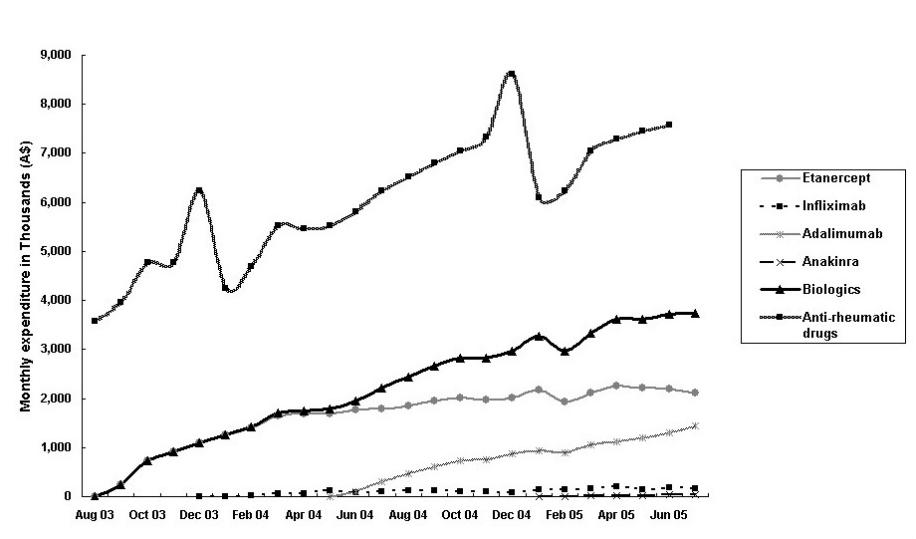
Monthly expenditure on anti-rheumatic drugs under the PBS and RPBS, August 2003–July 2005. Note: Expenditure on DMARDs was obtained from the Drug Utilization Sub-Committee database. DMARDs included: methotrexate, azathioprine, cyclophosphamide, cyclosporin, hydroxychloroquine, penicillamine, sulfasalazine, gold preparations (sodium aurothiomalate and auranofin), and leflunomide.

Reimbursed prescriptions for biologicals doubled in the second year accounting for 71% of total prescriptions over the two years. The government expenditure rose proportionally but was well below the "cap" of A$140 million per year (19%). There was only a marginal increase in the number of prescriptions for 'initial treatment' in the second year. The proportion of prescriptions for 'continuing treatment' increased from 50% to 72% of the total prescriptions in year 2 (Figure 1). Infliximab was not included in these estimates because prescriptions for this biological were not stratified into 'initiating' and 'continuing' categories. The maximum number of prescriptions processed in a month occurred in July 2005 – a total of 1,984 prescriptions for the biologic group (Figure 2) at a cost A$3.75 million (Figure 3). Assuming that each prescription was supplied to an individual patient, by crude estimation approximately 2,000 patients had commenced biological therapy for RA within the study period based on the aggregated data. This represents about half of the estimated 4,000 adult patients with RA predicted to be eligible for etanercept in the first year. However, it was not possible to determine the proportion of patients that were approved to continue or who were switched between biologicals.

The total number of prescriptions for etanercept under the PBS from August 2003 to July 2005 was 20,398 (A$38.4 million). A further 344 prescriptions (A$647,500) were reimbursed under the RPBS. Initiation of etanercept therapy declined after December 2003. The use of infliximab was considerably lower than etanercept, although infliximab was not PBS-subsidised until November 2003: 826 prescriptions were provided under the PBS (A$2.1 million) and only 25 prescriptions (A$59,000) under the RPBS. Adalimumab was available under the PBS from May 2004. Up to July 2005, adalimumab had accounted for 6,063 prescriptions (A$11.4 million) under the PBS and 194 prescriptions (A$365,800) under the RPBS. Since inclusion on the PBS in December 2004, anakinra had accounted for 115 prescriptions (A$164,000) under the PBS and 5 prescriptions (A$7,100) under the RPBS (Table 2).

**Table 2:** Prescriptions of biologicals for rheumatoid arthritis under the PBS and RPBS by Australian States and Territories based on aggregate data, Aug 2003–Jul 2005

	NSW	VIC	QLD	SA	WA	TAS	ACT	NT	TOTAL
Number of prescriptions									
Etanercept	7 559	3 685	2 205	3 064	2 142	1 085	939	63	20 742
Infliximab^a^	190	182	146	51	280	2	0	0	851
Adalimumab	1 842	1 496	1 096	503	820	369	116	15	6 257
Anakinra	52	17	25	8	17	0	1	0	120
Biologicals Total	9 643	5 380	3 472	3 626	3 259	1 456	1 056	78	27 970

Biologicals Prescriptions per 10,000 population^b^	14.4	10.9	9.0	23.7	6.6	30.3	32.7	3.9	
	
Number of rheumatologists^c^	86	69	32	29	24	5	5	2	
	
Rheumatologists per 10,000 population	0.128	0.139	0.083	0.189	0.122	0.104	0.155	0.100	
	
Biologicals Prescriptions per rheumatologist	112.1	78.0	108.5	125.0	135.8	291.2	211.2	39	

Note:

a Number of prescriptions of infliximab: usage in public hospital is not included in the statistical report by Medicare Australia.

b Source: Australian Bureau of Statistics, Australian Population – States and Territories, December quarter, 2003.

c Number of rheumatologists registered as members of the Australian Rheumatology Association

Australian states and territories: NSW = New South Wales, VIC = Victoria, QLD = Queensland, SA = South Australia, WA = Western Australia, TAS = Tasmania, ACT = Australian Capital Territory, NT = Northern Territory

### Prescriptions of biologicals by Australian states and territories

Uptake of biologicals by State/Territory was greatest in New South Wales (7,559 prescriptions). The relative *per capita *uptake demonstrated the same pattern as seen in the first year after listing of etanercept [23]. The Australian Capital Territory, Tasmania and South Australia had the highest relative *per capita *use of biologicals (32.7, 30.3 and 23.7 prescriptions per 10,000 population, respectively). Initiations and continuations of biological therapies were also highest in these states. The Northern Territory had the lowest *per capita *use (Table 2). There was an 8-fold range of ratios between the jurisdictions. After adjusting for state population, uptake of infliximab was the highest in Western Australia. South Australia and the Australian Capital Territory had the highest *per capita *ratio of rheumatologists, whereas Queensland had the lowest. Prescription rates per rheumatologist were highest in Tasmania and the Australian Capital Territory.

### Prescriptions of biologicals by rural, remote and metropolitan areas

Based on the DUSC dispensing data on etanercept, infliximab, and adalimumab (data was supplied for Aug 2003–Jun 2005; anakinra dispensing data were unavailable from DUSC at the time of this study), a majority of prescriptions (89.6%) were provided by prescribers in the metropolitan areas (capital cities and other metropolitan centres; Table 3). Regional population numbers were not available for population-adjusted analysis. Review by the prescriber major specialty indicated that only a small proportion of prescriptions (5.7%) was provided by immunologists.

**Table 3:** Prescriptions of biologicals by Rural, Remote, and Metropolitan Area (RRMA) classification system, based on DUSC data (Aug 2003–Jun 2005)

**RRMA**	Etanercept	Infliximab	Adalimumab	Biologicals Total	% of Total
Capital cities	18669	802	6396	25867	80.25
Other metropolitan centres^a^	2124	52	826	3002	9.31
Large rural centres^b^	1517	138	611	2266	7.03
Small rural centres^c^	635	5	165	805	2.50
Other rural areas^d^	44	0	17	61	0.2
Remote areas^e^	22	0	13	35	0.11
Other remote areas^f^	4	1	0	5	0.02
Unknown	164	1	28	193	0.6

a Other metropolitan centres: population > 100 000 [27]

b Large rural centres: population 25 000 to 99 999

c Small rural centres: population 10 000 to 24 999

d Other rural areas: population < 10 000

e Remote areas: population > 5000

f Other remote areas: <5000

## Discussion

The utilisation and expenditure on biologicals for treating RA under the PBS over the first two years was substantially below that forecast (19% by expenditure). Uptake of etanercept was considerably lower than projected (14% by expenditure) in the first year [23] and the uptake rate did not increase significantly in the second year (Figure 1). Therefore, concerns about inappropriate or over-use of biologicals appear to be unfounded. From this perspective, the PBS restrictions have been effective in controlling access and containing government expenditure. By July 2005, approximately 1% of the RA patient population in Australia had commenced biological therapy. This is substantially lower than that reported in other countries, for example, 14.9% of patients with RA were treated with biologicals in southern Sweden in 2003 [28], and about 20% of patients with RA receive anti-TNF therapy in the United States [29]. It needs to be acknowledged that the number of patients and hence the expenditure on biologicals are likely to vary with the eligibility criteria for the treatment [30]. The low use of biologicals in Australia may reflect, in part, the administrative burden imposed by the PBS restrictions, which may have discouraged some applications. Administrative tasks may have also contributed to the lower than expected registration of patients on the voluntary national biologicals registers established to track patient outcomes [31]. Another possible explanation is that there has been cautious selection of patients by rheumatologists because of concerns about drug safety. It is also possible that a smaller population of RA patients than predicted achieved the eligibility criteria for initiating biological therapy as a result of the PBS-mandated treatment algorithm (including the use of combination DMARD therapy). The non-biologicals regimen may have clinically accommodated a greater proportion of patients than expected such that criteria indicating substantial disease activity necessary for access to biologicals were no longer met. The true value of this algorithm  needs to be reviewed because evidence supports a conclusion that biologicals  are more efficacious than DMARDs in arresting joint damage  [32]. Withdrawal from biological therapy may also explain lower prescribing and expenditure than expected. It has been reported that up to 40% of patients do not or only partially respond to biologicals [33]. Finally, it is also likely that the forecasts were inaccurate; data assembled by pharmaceutical companies including the economic analyses have been reported to be error prone [34]. Both under- and over-estimates of usage compared with actual usage of other drugs have been reported in two-thirds of submissions for subsidy of medicines made to the PBAC [35]. Summaries on submissions for drug subsidy, including the estimation of expected usage through the PBS and the cost to government, and the outcomes of PBAC decisions are now published [36].

Etanercept had the highest usage over the study period. This finding was expected because etanercept was the first agent available under the PBS (August 2003). Further, patients who were intolerant to methotrexate could only be treated with etanercept. Adalimumab was more recently approved as monotherapy in April 2005. The gradual plateau in the utilisation of etanercept (Figure 2) possibly reflects the introduction of adalimumab under the PBS. The steady increase in initiations of adalimumab treatment may reflect the fortnightly administration schedule as compared with etanercept that is administered twice per week. The considerably lower use of infliximab is possibly due to several reasons: it must be used in combination with methotrexate, it is used less frequently (administered every 6–8 weeks), its usage in public hospitals is not captured, and it is administered as an intravenous infusion which may be much less preferred by prescribers and patients. There has been no contrast in efficacy outcomes between TNFIs demonstrated to date in RA. The minimal use of anakinra can be explained by its recent listing on the PBS (December 2004) and data that suggest it is less efficacious than TNFIs [15]. The rate of uptake of anakinra over the next few years is  not easily predicted because patients who fail to respond to two TNFIs are allowed to trial anakinra (under the 'interchangeability rule').

An important goal of the PBS is to provide equitable access to medicines for the community. However, those in equal need may not have equal opportunities to access rheumatological services. Prescribing of these drugs under the PBS is restricted to rheumatologists and clinical immunologists with expertise in the management of RA. There is a limited number of specialists and reasonable access to a rheumatologist varies considerably between states and territories in Australia. Not surprisingly the use of biologicals roughly correlated with the *per capita *ratio of rheumatologists. Patients in remote or rural areas are thus disadvantaged by this criterion. Prescription data by rural, remote and metropolitan areas also indicated geographical heterogeneity in access to health care. Other factors potentially influencing the use of specialist care include patient's income, indirect costs (e.g. travel costs, foregone wages), access to information, knowledge, and cultural beliefs [37]. Geographical variation in access to biologicals has also been identified in the United Kingdom ('post code prescribing') [38].

The PBS expenditure on biologicals increased steadily over the study period (Figure 3). Essentially the cost of biologicals is covered by the government (99.5%) with a minimal proportion contributed by patients through co-payments. The majority of expenditure on prescriptions of biologicals was directed towards concessional patients. This is not surprising as the PBS restrictions select for those patients with severe RA who are likely to suffer from functional disability; loss of work capacity in RA patients has been well recognised [39]. Monthly PBS expenditure on anti-rheumatic drugs overall (including both DMARDs and the biologicals) has doubled since the biologicals became available; although it should be acknowledged that not all usage of medicines classified as DMARDs is for RA. The relatively high cost of biologicals and the increasing expenditure on medicines needs to be examined together with other costs such as cost of monitoring and treating adverse reactions as well as the cost of managing RA [40] (direct costs such as other health services, indirect costs such as work disability, and intangible costs such as pain, fatigue, and psychological distress). The potential that these treatments may obviate the need for surgery associated with joint destruction and progressive functional disability characteristic of inappropriately controlled RA (cost savings) needs also to be taken into consideration [41]. The disease is associated with considerable socio-economic impact on individual patients and their families as well as on society as a whole. An estimated A$246 million spent on health services including 19% on pharmaceuticals (prescribed and over-the-counter medications) in the year 2000–01 was attributable to RA [18]. These data are concordant with a previous report that 8–24% of the direct cost of treating RA could be attributed to the cost of drugs [42]. The use of biologicals may influence the use of other services. For example, a small study in the United Kingdom reported a 55% reduction of in-patient services use while the use of out-patient services doubled [43], a factor which also needs to be examined. Further, the resources needed to administer the PBS criteria are expensive because of the complexity of the process [44]. Formal review of these costs and outcomes is important to an understanding of the value of such controlled access schemes for medicines.

There are several limitations of this analysis. The present study emphasises an urgent need for access to comprehensive data that would enable a more detailed examination of the use of medicines in Australia. The data discussed here were derived from the Medicare Australia national administrative database, which is available through its website [22]. De-identified, individual data on patient demographics and usage of medicines can be purchased from Medicare Australia, but is not readily accessible. Data on health outcomes are also unavailable, despite abundant information on clinical status being collected for approval of access to biologicals [45]. Further, data on the use of medicines, adverse drug reactions, and use of medical services are not linked or easily available for linkage in Australia. Utilisation of medicines and medical services in public hospitals, managed by state governments, are also not incorporated in the national dataset. Australia lags behind other countries in this area e.g. the General Practice Research Database in the United Kingdom [46], and national registries established for anti-rheumatic treatments in several countries [31,47]. These databases hold more comprehensive datasets that can potentially identify and address important issues, including utilisation of medicines, and access to and quality of health care. Developments and successes with state-based data linkage of Commonwealth and state data such as that established in Western Australia [48] have been encouraging and will enable individual-level studies of medicine use and associated health outcomes. Other Australian States, including New South Wales, Queensland and South Australia are currently establishing mechanisms for linkage between State and Commonwealth health data [49].

A recent report of an increased risk of serious infections, a dose-dependent increased risk of malignancies in patients with RA treated with biological therapies [50], as well as the expanded clinical uses of these drugs (such as in the treatment of psoriatic arthritis and ankylosing spondylitis) also emphasise the need for ongoing surveillance. Evaluation based on comprehensive data would enable clearer conclusions to be reached about the clinical and economic effects of the PBS controlled access scheme for biologicals, specifically, the initial access criteria, the 'continuation rule', and the 'interchangeability rule'. This feedback is essential for decision makers to refine the criteria and for prescribers to manage patients appropriately and cost-effectively.

## Conclusion

Utilisation of high-cost, anti-rheumatic biologicals in Australia over the first two years of PBS subsidy has been conservative, well below the forecast. Whether this low uptake represents appropriate or inappropriate prescribing of biologicals in Australia cannot be determined on the basis of the limited available information. Recent increased transparency on how projections of utilisation and expenditure on medicines are determined is to be applauded. Ongoing review of PBS restrictions as new data become available together with post-subsidy examination of drug utilisation, associated costs, and resultant health outcomes, is important. Enhanced communication between all parties will increase confidence in the drug subsidy program and its decisions, and hence its sustainability as a public system. These enhancements will ensure that there is affordable, equitable, and efficient provision of needed medicines to the individual patient with resultant improved health outcomes being delivered by a national public reimbursement system.

## Competing interests & Funding/support

Christine Lu was supported by an Australian National Health and Medical Research Council postgraduate research scholarship (Grant No. 351040). A/Prof Ken Williams has been a member of the Advisory Board for the sponsor of adalimumab. Prof Ric Day is a member of Advisory Boards for sponsors of adalimumab, infliximab, and anakinra. A/Prof Williams and Prof Day have also been contracted to undertake clinical trials of etanercept, infliximab, adalimumab, and anakinra. Recompense for these activities was placed in audited hospital trust funds for use in the research activities of the Clinical Pharmacology Department, St Vincent's Hospital, Sydney.
